# Multidimensional Digital Literacy and Quality of Life Among Informal Care Dyads in Malaysia: Cross-Sectional Survey

**DOI:** 10.2196/86561

**Published:** 2026-04-27

**Authors:** Fereshteh Mohammadzadeh Yazd, Hui Foh Foong, Rahimah Ibrahim, Puvaneswaran Kunasekaran, Asmidawati Ashari

**Affiliations:** 1 Malaysian Research Institute on Ageing (MyAgeing™), Universiti Putra Malaysia, UPM Serdang, 43400, Malaysia, 60 3-9769 2755; 2 Faculty of Human Ecology, Universiti Putra Malaysia, Serdang, Malaysia

**Keywords:** digital literacy, quality of life, older adults, care recipients, caregivers, informal caregiving, caregiving dyads, dyadic research, actor-partner interdependence model, Malaysia

## Abstract

**Background:**

Digital literacy (DL) is a key determinant of health and social participation in later life. In Malaysia, where population aging and family caregiving are rising, limited digital engagement among older adults may widen the gray digital divide. As caregivers and care recipients are interdependent, their digital capacities may jointly shape each other’s quality of life (QoL).

**Objective:**

This study examined the multidimensional associations between DL and QoL among informal caregivers and care recipients in Malaysia and explored dyadic actor-partner effects.

**Methods:**

A cross-sectional dyadic survey was conducted with 72 caregiver-care recipient dyads (N=144) recruited from 4 sites across Klang Valley, Malaysia, through purposive sampling. Dyads were the unit of analysis. DL was measured using the 22-item Everyday Digital Literacy Questionnaire across 3 dimensions (information and communication, content creation and management [CCM], and safety and security [SS]), while QoL was assessed with the World Health Organization Quality of Life-BREF. Partial least squares structural equation modeling tested dimension-specific links, and the actor-partner interdependence model examined bidirectional effects.

**Results:**

Caregivers reported higher DL and QoL than care recipients. Among caregivers, only “SS” significantly predicted QoL (*β*=.44, *P*<.001); among care recipients, all 3 dimensions were significant, with “CCM” showing the strongest effect (*β*=.53, *P*<.001). The actor-partner interdependence model indicated significant actor and partner effects (caregiver actor *β*=.53, partner *β*=.34; care recipient actor *β*=.60, partner *β*=.45; all *P*<.001), explaining 62% and 60% of variance in QoL.

**Conclusions:**

DL shows a clear actor-partner pattern. For actor effects, caregivers’ QoL was linked to their own SS skills, while care recipients’ QoL was linked to their own CCM skills. Partner effects showed that care recipients’ CCM skills also predicted caregivers’ QoL. Both effects were significant, indicating interdependence within the caregiving dyad. These findings can inform dyad-focused and dimension-specific DL interventions to enhance mutual well-being.

## Introduction

Population aging is a global phenomenon. It is transforming social, economic, and health systems worldwide [1]. Increasing longevity and declining fertility rates have led to a larger proportion of older adults in the population. This has caused reliance on informal caregivers, such as spouses or relatives, who provide unpaid care at home [2]. Meanwhile, caregivers have a pivotal role in maintaining the health and well-being of older adults. However, they often experience heavy burdens, such as physical strain, emotional stress, and financial burden, which reduce their quality of life (QoL) [3]. Research has shown that the health and well-being of caregivers and care recipients are interdependent. Emotional distress in one often mirrors the experience of the other [4,5]. This interdependence highlights that we should seek digitally inclusive solutions to improve the well-being of both caregivers and care recipients. In Asia, this need is even more crucial with the rapid growth of the aging population and the spread of digital technologies.

Population aging is progressing rapidly in Asia [6], and the need for long-term and family-based care systems is increasing [7]. Malaysia is an example of this demographic shift, with an estimated 15% of the population projected to be over 60 years of age by 2030 [8]. However, the digital engagement and participation of older adults are still limited. A recent study in Malaysia highlighted that while device ownership is increasing, actual utilization, specifically for health information, remains low (27%). Furthermore, a majority of older adults (68%) perceive online information as unreliable or lack the necessary confidence and skills to navigate digital platforms effectively [9]. These figures indicate a persistent “digital gray divide.” This means that inequalities in access, trust, and digital skills among older adults persist [10]. As family-based caregiving is still the main form of supporting older adults in Malaysia [11], strengthening digital skills within these caregiving relationships is essential.

Digital literacy (DL) is the ability to find, evaluate, and use digital information effectively [12]. It has been identified as a major determinant of health and care outcomes [13]. DL goes beyond technical skills. It helps people access online health care services, communicate with health care professionals, and use digital resources for their daily living. Higher DL is associated with lower caregiver stress and better QoL for both caregivers and care recipients [14,15]. A recent scoping review shows that informal caregivers with stronger digital skills experience greater empowerment. As a result, they feel confident in their caregiving role [16]. Yet, a lack of DL is associated with limited access to reliable information. It is linked to increased vulnerability to misleading information and may exacerbate inequalities in health participation [17,18]. This gap not only affects how caregivers search for information but also how they support care recipients.

Malaysia’s aging policies have identified technology as a tool to enhance independence and achieve “supported aging” [19]. However, the National Policy for Older Persons [19], which was developed in the early 2000s, was formulated at a time when concepts such as “digital literacy” and “digital inclusion” were not yet embedded in social policy-making. For this reason, this document does not explicitly address digital participation or the role of informal caregivers in technology use and adoption [20]. Similarly, the Malaysia Digital Economy Blueprint [21] focuses more on innovation, economic growth, and industrial transformation. Although the document emphasizes the need for DL, it does not pay much attention to the specific needs of care recipients and caregivers. Overall, despite the growing attention to technology in existing policies, a comprehensive framework for integrating care recipients and caregivers into the country’s digital ecosystem has not yet been formed [16].

Recent studies suggest that digital skills can be associated with increased autonomy, social participation, and health-promoting behaviors among care recipients and caregivers [7,22]. Targeted training programs have also been shown to improve digital skills and self-efficacy in these groups [23,24]. In dyadic caregiving relationships, the caregiver’s use of technology is associated with the care recipient’s level of digital participation [25], such that higher DL in one person is associated with higher DL in the other. Although several recent studies have applied dyadic approaches to examine health literacy or eHealth literacy in patient-caregiver dyads [26,27], these investigations have generally focused on health behaviors or technology use intentions rather than on everyday DL or QoL. Consequently, little is known about how multidimensional DL shapes actor-partner patterns of QoL within informal caregiving relationships.

Given the distinct roles and digital engagement patterns of caregivers and care recipients, we expected that different DL dimensions might show differential importance for each group. For caregivers, who frequently seek health information online on behalf of care recipients, safety and security (SS) skills may be particularly salient in reducing exposure to misinformation and digital risks [16,28]. For care recipients, communication and content creation skills may be more strongly linked to QoL, as these dimensions enable active social participation and self-expression, key components of autonomy and social connectedness in later life [29,30].

Building on this evidence and the concept of interdependence in caregiving relationships [4,31], this study examines how multidimensional DL, including the dimensions of “information and communication (IC),” “content creation and management (CCM),” and “SS,” is related to the QoL of informal caregivers and care recipients in Malaysia. Using the actor-partner interdependence model (APIM), this study seeks to test the hypothesis that the DL of each member of a caregiving dyad is related not only to their own QoL (actor effect) but also to the QoL of their partner (partner effect). It is expected that examining this model will lead to a more comprehensive understanding of the multidimensional role of DL in promoting the mutual well-being of caregivers and care recipients and provide a basis for future research and intervention programs in the field of digital aging.

## Methods

### Study Design, Setting, and Participants

This study was a cross-sectional, dyadic design conducted in the Klang Valley region of Malaysia (Selangor and the Federal Territory of Kuala Lumpur) between May and October 2025. Eligible participants were informal caregivers aged 18 years and older and community-dwelling older adults aged 60 years and older (hereinafter referred to as “care recipients”) residing in the urban Klang Valley region. The caregiver had to be the primary support person nominated by the care recipient, and both were required to complete the questionnaire independently. Informal caregiver-care recipient dyads were the unit of analysis.

A pilot study with 20 dyads was conducted to estimate the population parameters for sample size determination. To address the dyadic nature of the data, a Monte Carlo power analysis was performed using Mplus Version 8.3 [32], following guidelines for sample size estimation in structural equation modeling (SEM) [33]. Based on pilot results and dyadic research recommendations, we hypothesized standardized actor effects of 0.50 and partner effects of 0.37. The simulation parameters were set at *α*=.05 with 1000 replications using the maximum likelihood robust (MLR) estimator. Results indicated that a sample size of 69 dyads would achieve a statistical power of greater than 0.80 to detect both actor and partner effects. Participants were recruited from 4 sites, including community centers, care service organizations, and public events across Klang Valley, through purposive sampling. A total of 100 dyads were approached and consented. As expected in dyadic field research, some dyads were incomplete because one member did not return the questionnaire, in which case the entire dyad was excluded. Ultimately, 72 complete dyads were obtained (72% dyad-level participation), exceeding the a priori target and falling within the sample size range often recommended for stable estimation in APIM studies [34].

To ensure reliable self-reports, participants were screened for subjective memory complaints using a single-item cognitive self-assessment. This brief approach is supported by previous research showing that the absence of memory complaints is a useful indicator of adequate cognitive ability for independent questionnaire completion [35]. No dyads were excluded based on this screening item.

### Measures

#### Demographic Characteristics

Demographic characteristics were collected for both caregivers and care recipients, including age, gender, marital status, ethnicity, education level, employment status, and monthly household income. Information on internet access (frequency, connection quality, and device use) and self-rated health status was also obtained.

#### Digital Literacy

DL was assessed using the 22-item Everyday Digital Literacy Questionnaire (EDLQ) [36]. The instrument is conceptually grounded in the European Commission’s DigComp framework and broader multidimensional conceptualizations of DL. It was validated with a 3-factor structure. Each item is rated on a 5-point Likert scale ranging from 1 (“Never True”) to 5 (“Always True”), with higher scores indicating greater DL.

**Figure 1.**
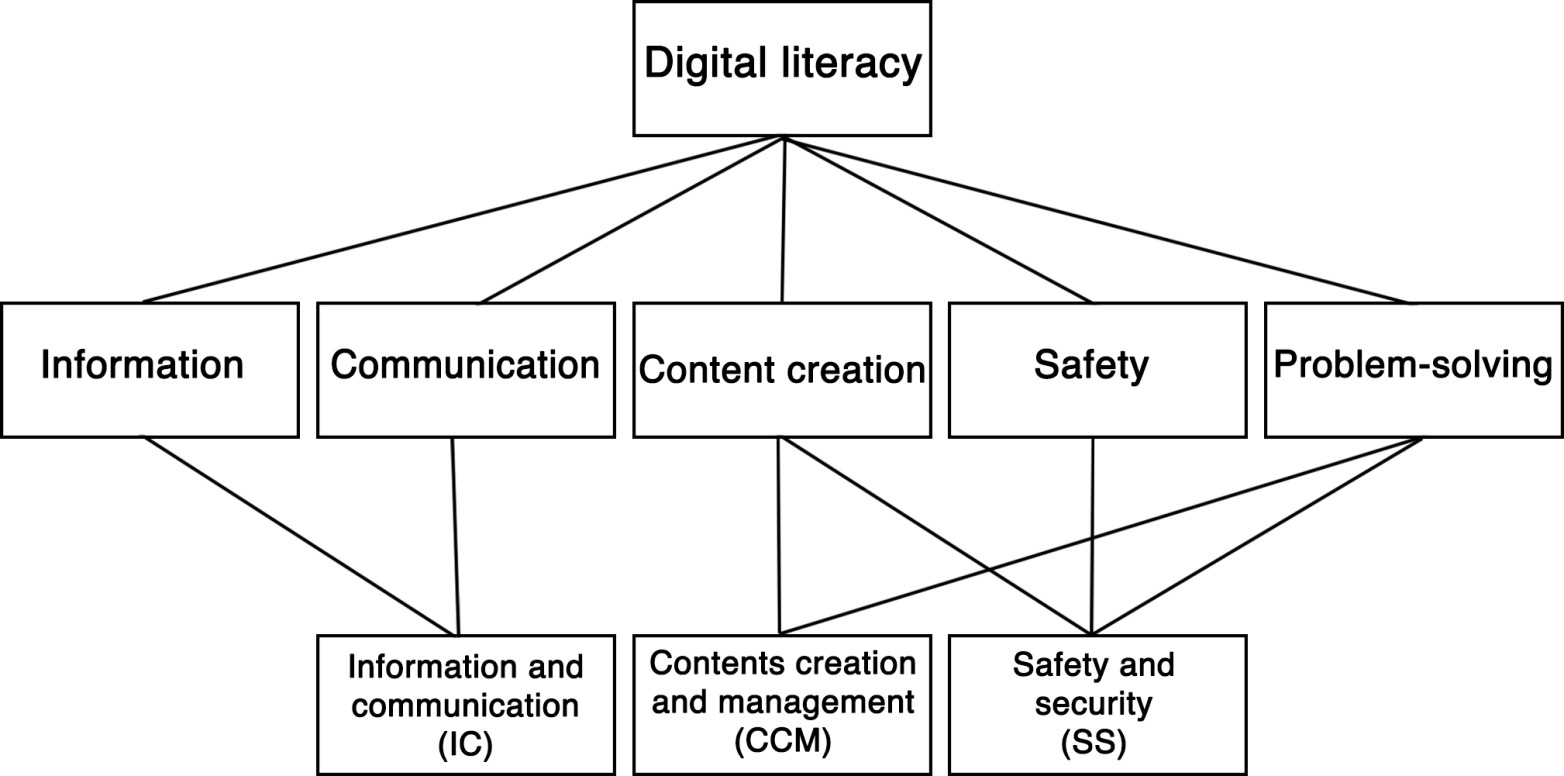
The Everyday Digital Literacy Questionnaire structure, adapted from Choi et al [36].

Following the authors’ scoring guidelines, 3 domain scores were calculated: IC (mean of DL01-DL09), CCM (mean of DL10-DL13), and SS (mean of DL14-DL22). In addition, a total score (sum of all 22 items, range 22‐110) was computed to capture overall DL. Scores were calculated separately for caregivers and care recipients. The EDLQ was originally developed and validated in Korean. English versions of all 22 items were available in the original validation study [36]. The English items were translated into Malay and reviewed by a bilingual academic with expertise in gerontology for linguistic clarity and cultural appropriateness. Due to sample size constraints, measurement invariance testing across languages was not conducted and is acknowledged as a limitation. The 3 domains of the EDLQ are illustrated in Figure 1.

#### Quality of Life

QoL was assessed using the World Health Organization Quality of Life-BREF (WHOQOL-BREF), a 26-item instrument developed by the World Health Organization to measure individuals’ perceived QoL across 4 domains: physical health, psychological health, social relationships, and environment [37,38]. Each item is rated on a 5-point Likert scale with varying anchors (eg, 1=“very poor” to 5=“very good,” or 1=“not at all” to 5=“completely”), reflecting the intensity, capacity, frequency, or evaluation of life experiences during the past 2 weeks. Higher scores indicate better QoL. Domain scores were computed following WHO scoring guidelines and transformed to a standardized 0 to 100 scale using the recommended formula: (transformed score=[raw score–minimum possible score]×100/[maximum possible score–minimum possible score]) [37].

An overall QoL index was calculated by averaging the 4 domain scores. In this study, the 4 WHOQOL-BREF domains showed very high intercorrelations (caregivers: *r*=0.851-0.928; care recipients: *r*=0.863-0.938; all *P*<.001), indicating substantial shared variance and supporting the use of a composite measure of overall well-being [38]. Moreover, examining all 4 domains separately would require multiple independent models (raising concerns about statistical power and Type I error inflation with N=72 dyads). Therefore, this composite score was used for subsequent SEM and APIM analyses. Scores were analyzed separately for caregivers and care recipients.

### Ethical Considerations

Data were collected through structured, self-administered questionnaires. The questionnaires were available in both English and Malay, and participants completed the version in their preferred language. Each dyad received 1 set of questionnaires, and an online completion option was also provided. Responses were checked to ensure caregiver-care recipient matching.

Institutional Review Board approval was granted by the Ethics Committee for Research Involving Human Subjects (JKEUPM) of Universiti Putra Malaysia (approval number: JKEUPM-2025-037). Participation was voluntary, and written informed consent was obtained from all respondents. Confidentiality was ensured through anonymized data, and participants could withdraw at any stage without consequence. Participants were not compensated for their participation in this study.

### Statistical Analysis

Statistical analyses for this cross-sectional dyadic data were conducted to describe participant characteristics, validate the measurement model, and examine dyadic actor-partner effects of DL on QoL.

Descriptive statistics (means, SDs, frequencies, and percentages) were computed in IBM SPSS Statistics Version 26. Construct validity and reliability of the measurement model were evaluated in SmartPLS Version 4.0.9.6 for both the EDLQ and WHOQOL-BREF. Reliability and convergent validity were confirmed based on item loadings ≥0.70, Cronbach α and composite reliability >0.70, and average variance extracted >0.50. Discriminant validity was established via the heterotrait-monotrait ratio <0.85. Collinearity was assessed using the variance inflation factor (VIF<3.6). Model fit was assessed using standardized root mean square residual (SRMR=0.069 for caregivers; 0.075 for care recipients). Path significance was tested using bootstrapping with 5000 resamples, and predictive relevance (Q²=0.429 for caregivers; 0.499 for care recipients) was examined via blindfolding.

To test dyadic relationships, the APIM was estimated in Mplus Version 8.3 using MLR, consistent with established applications of dyadic data analysis [32,39,40]. Dyads were treated as distinguishable (caregiver vs care recipient). Because these roles are theoretically and structurally nonexchangeable, an empirical distinguishability test was not required [41]. Missing data were handled using full-information maximum likelihood (FIML), which used all available data under the missing-at-random assumption. In the APIM variables, there were no missing data for the DL predictors, whereas QoL outcomes had 25% (18/72) missing values for caregivers and 18% (13/72) for care recipients.

To assess the robustness of FIML estimates, 3 sensitivity analyses were conducted: Little MCAR test to verify the missing-at-random assumption, expectation-maximization (EM) imputation to assess potential bias from missingness, and APIM re-estimation using listwise deletion (n=47 complete dyads) to compare with FIML results. These analyses were performed in IBM SPSS Statistics 26 (Little MCAR, EM imputation) and Mplus 8.3 (listwise deletion APIM).

It is important to note that while APIM permits the examination of actor and partner effects, the cross-sectional design limits the ability to infer causality or temporal precedence. The APIM framework is used here to model patterns of association and interdependence within dyads at a single time point, consistent with standard applications in cross-sectional dyadic research [34].

The APIM accounts for actor effects (an individual’s DL → their own QoL) and partner effects (one member’s DL → the other’s QoL). Residuals of the 2 QoL outcomes were allowed to covary. Standardized coefficients (*β*), standard errors, 95% CIs, and explained variance (*R²*=0.619 for caregivers; 0.595 for care recipients in the basic model) were reported. Because both APIM specifications (basic and multivariate) were saturated (*df*=0), global fit indices (Comparative Fit Index, Tucker-Lewis Index, root mean square error of approximation, SRMR) were not interpreted, and modification indices were not applicable. Model evaluation focused on path coefficients and explained variance.

Two APIM specifications were estimated. The basic model used each member’s composite DL score to predict their own QoL (actor effect) and their partner’s QoL (partner effect). Composite scores were used in the model because latent APIM requires substantially larger samples for stable estimation, and APIM is commonly estimated using observed scores in small to moderate samples [39,41]. Additionally, the high reliability of the measures (care recipient >0.90), as shown in Table 4, supports the use of composite scores as valid proxies for the latent constructs. The multivariate model simultaneously included the 3 EDLQ dimensions (IC, CCM, and SS) as predictors of QoL, allowing estimation of domain-specific actor and partner effects while retaining dyad distinguishability and correlated residuals. All analyses were 2-tailed, and statistical significance was set at *P*≤.05.

## Results

### Participant Characteristics

**Figure 2.**
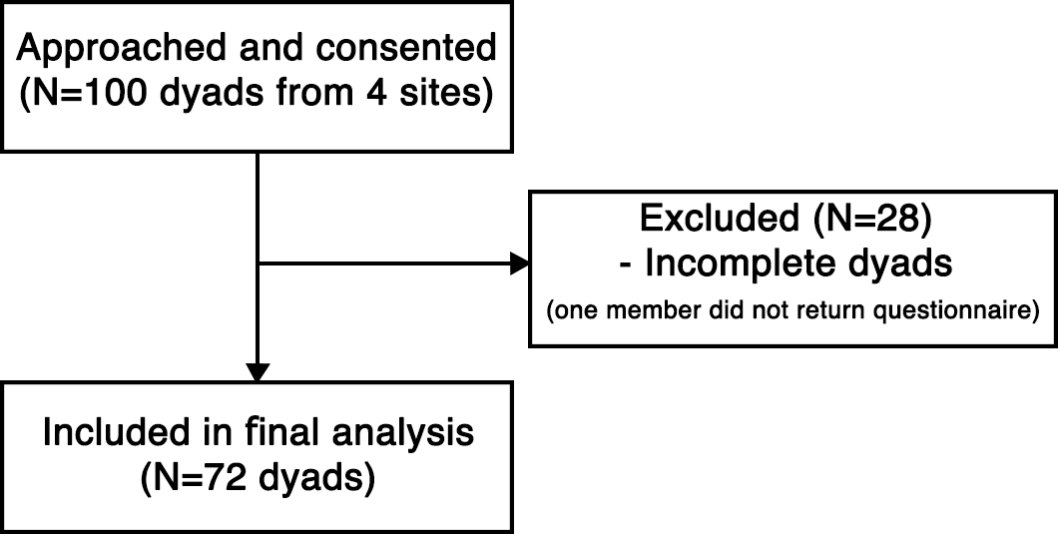
Flowchart of participant recruitment and selection process.

Figure 2 presents the participant recruitment and selection flow. Of the 100 dyads approached, 72 caregiver-care recipient dyads were included in the analysis. The mean age of caregivers was 51 (SD 7.4) years and that of care recipients was 75.3 (SD 4.5) years. Most caregivers were female (43/72, 60%). Among care recipients, 57% (41/72) were female. The sample was predominantly Malay, followed by Chinese and Indian participants. Educational levels were generally higher among caregivers than care recipients. About one-third of caregivers had a bachelor’s degree or higher, compared to 11% (8/72) of care recipients. Caregivers reported better internet access than care recipients, more than half of whom had weak access. Most caregivers rated their health as good, whereas only one-third of care recipients reported good health. Table 1 presents the characteristics of caregivers and care recipients.

**Table 1.** Participant characteristics of caregivers and care recipients (N=72 dyads).

Characteristics	Caregivers	Care recipients
Age (y), mean (SD; range)	51 (7.4; 37‐65)	75.3 (4.5; 61‐85)
Gender, n (%^a^)		
Female	43 (60)	41 (57)
Marital status, n (%)		
Married	45 (71)	42 (64)
Widowed	13 (21)	24 (36)
Ethnicity, n (%)		
Malay	38 (57)	36 (55)
Chinese	15 (22)	15 (23)
Indian	14 (21)	14 (22)
Education level, n (%)		
High (≥bachelor’s degree)	25 (35)	8 (11)
Low (≤secondary)	22 (31)	44 (61)
Internet access, n (%)		
Strong	33 (46)	1 (1)
Moderate	22 (31)	32 (44)
Weak	17 (24)	39 (54)
Good self-rated health, n (%)	42 (58)	25 (35)

a Percentages may not sum to 100% due to rounding and missing data.

### Descriptive Statistics and Correlations

Table 2 presents the descriptive statistics for the main study variables. Caregivers reported moderate levels of DL across the 3 dimensions (mean range 3.4‐3.6; Table 2). Care recipients showed slightly lower scores, particularly for CCM. Caregivers had higher overall QoL scores than care recipients, and because care recipient QoL was nonnormally distributed, median (IQR) is also reported.

**Table 2.** Descriptive statistics of caregivers and care recipients (N=72 dyads).

Variables	Mean (SD)	Minimum-maximum	Median (IQR)
Caregivers			
Information and Communication	3.47 (0.74)	1.63-5.00	—^a^
Content Creation and Management	3.44 (0.76)	1.25-5.00	—
Safety and Security	3.60 (0.78)	1.75-5.00	—
Overall QoL^b^ (0‐100)	64.46 (14.68)	37.50-97.28	64.36 (20.45)^c^
Care recipients			
Information and Communication	3.23 (0.78)	1.38-4.86	—
Content Creation and Management	2.57 (0.70)	1.00-4.00	—
Safety and Security	3.17 (0.72)	1.43-4.71	—
Overall QoL (0‐100)	55.27 (16.34)	19.01-84.84	60.26 (21.35)

a Not applicable.

b QoL: quality of life.

c Median and IQR are reported for QoL variables.

The 3 DL dimensions were positively intercorrelated within each group. All DL dimensions were significantly and positively correlated with QoL within each group (all *P*<.001). Cross-dyad correlations also emerged: caregivers’ DL dimensions were positively associated with care recipients’ QoL, and vice versa. These preliminary results suggest potential interdependence between caregiver and care recipient DL and QoL, supporting the rationale for subsequent APIM analyses. Pearson correlations among study variables (computed using pairwise deletion) are shown in Table 3.

**Table 3.** Pearson correlation coefficients among digital literacy dimensions and QoL^a^ (N=72 dyads).

Variable	1	2	3	4	5	6	7	8
CG^b^ information and communication	—^c^	0.57	0.64	0.18	0.56	0.08	0.63	0.48
CG content creation and management	0.57	—	0.53	0.12	0.46	0.19	0.59	0.56
CG safety and security	0.64	0.53	—	0.15	0.62	0.13	0.68	0.48
CR^d^ information and communication	0.18	0.12	0.15	—	0.34	0.62	0.49	0.59
CR content creation and management	0.56	0.46	0.62	0.34	—	0.25	0.96^e^	0.68
CR safety and security	0.08	0.19	0.13	0.62	0.25	—	0.40	0.49
CG QoL	0.63	0.59	0.68	0.49	0.96^e^	0.40	—	0.74
CR QoL	0.48	0.56	0.48	0.59	0.68	0.49	0.74	—

a QoL: quality of life.

b CG: caregiver.

c Not applicable.

d CR: care recipient.

e The correlation between CR content creation and management and CG QoL (0.96) reflects the subsample with complete data (n=54). Note that the primary actor-partner interdependence model analysis uses FIML (full-information maximum likelihood; n=72) to handle missingness, yielding a robust but lower standardized estimate (*β*=.83) as reported in Table 7. A scatter plot confirming variable distinctness is provided in Multimedia Appendix 1.

### Measurement and Partial Least Squares SEM

The measurement model demonstrated satisfactory reliability and validity. All constructs demonstrated adequate reliability and convergent validity (Table 4). Discriminant validity was established using heterotrait-monotrait ratio criteria (all values <0.85). VIF values indicated no multicollinearity among constructs (all VIF <3.6). Model fit was acceptable for both caregiver and care recipient models (SRMR=0.069 for caregivers; 0.075 for recipients).

**Table 4.** Construct reliability and convergent validity of measurement model (N=72 dyads).

Group and construct	Cronbach α	Composite reliability	AVE^a^
Caregivers			
CCM^b^	0.89	0.92	0.75
IC^c^	0.94	0.95	0.66
SS^d^	0.95	0.96	0.71
QoL^e^	0.95	0.97	0.87
Care recipients			
CCM	0.86	0.91	0.71
IC	0.93	0.94	0.63
SS	0.91	0.93	0.58
QoL	0.96	0.97	0.90

a AVE: average variance extracted.

b CCM: content creation and management.

c IC: information and communication.

d SS: safety and security.

e QoL: quality of life.

**Figure 3.**
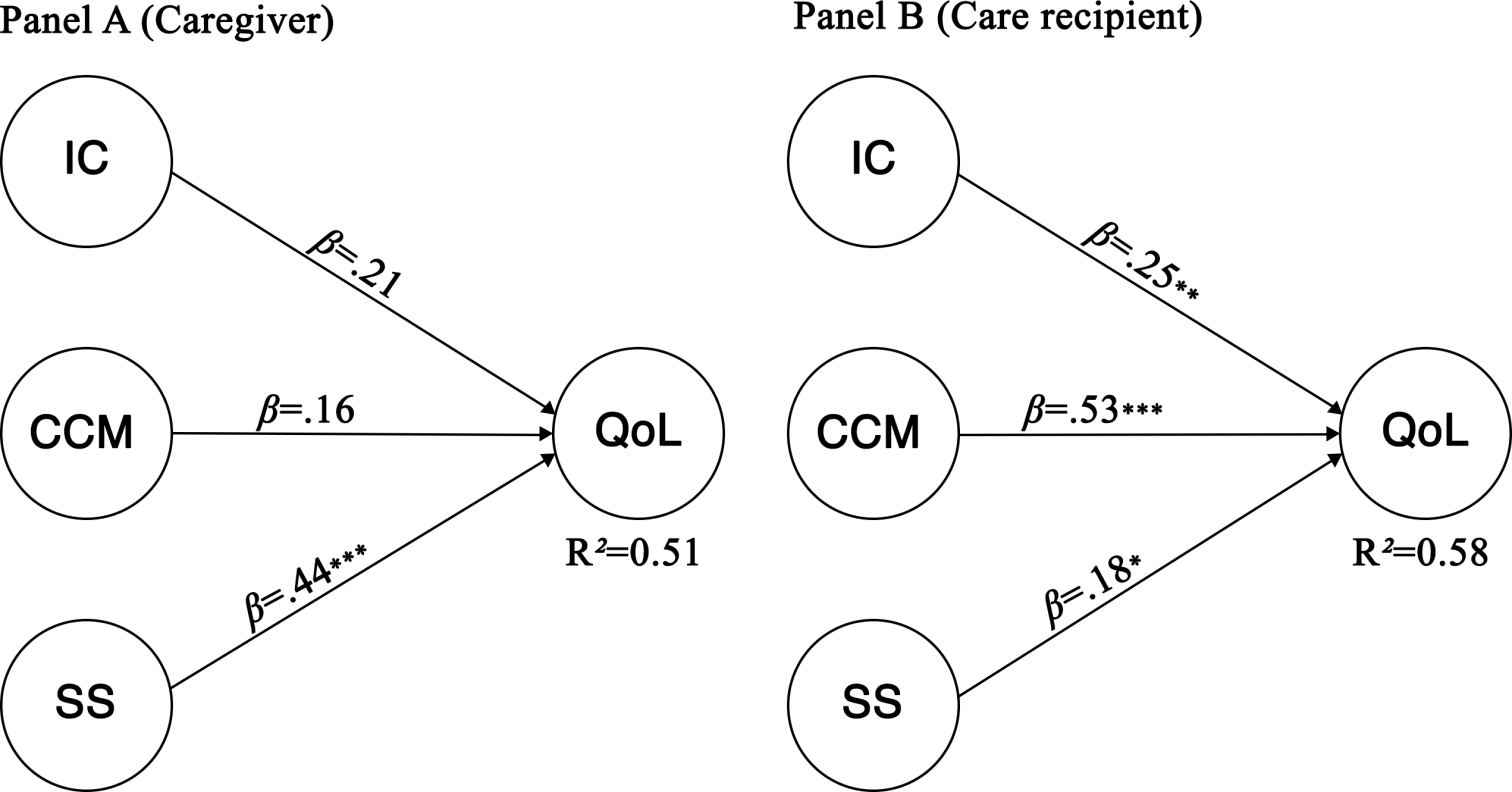
Structural partial least squares structural equation modeling models for caregivers (panel A) and care recipients (panel B), showing standardized path coefficients (*β*) and explained variance (R²). N=72 dyads. CCM: content creation and management; IC: information and communication; QoL: quality of life; SS: safety and security. *P<.05, **P<.01, ***P<.001.

The structural model explained 51% (37/72) of the variance in caregivers’ QoL and 58% (42/72) of the variance in care recipients’ QoL. Predictive relevance was supported for both models. Among caregivers, only the SS dimension significantly predicted QoL (*β*=.44, *P*<.001), whereas other dimensions were not significant. For care recipients, all 3 dimensions significantly predicted QoL, with CCM showing the strongest effect (*β*=.53, *P*<.001). Effect size analyses supported these patterns, indicating large effects for CCM among care recipients and for SS among caregivers. Figure 3 shows the structural partial least square-SEM models with standardized path coefficients and explained variance (*R²*) for caregivers (panel A) and care recipients (panel B). Full results are displayed in Table 5.

The partial least squares-structural equation modeling analyses established the validity of the measurement model and identified dimension-specific associations between DL and QoL within each role. These analyses treated caregivers and care recipients as independent groups. To examine bidirectional interdependence between dyad members as a central feature of caregiving relationships, we next employed the APIM, which explicitly accounts for the nonindependence of paired observations and tests whether each member’s DL predicts both their own (actor effect) and their partner’s (partner effect) QoL.

**Table 5.** Structural model results from partial least squares-structural equation modeling (bootstrapping).

Group and path	*β*	*t*	*P* value	*R²* (QoL^a^)	f²	Q²
Caregivers						
CCM^b^ → QoL	0.16	1.94	.052	0.51	0.03	0.43
IC^c^ → QoL	0.21	1.83	.07	—^d^	0.05	—
SS^e^ → QoL	0.44	4.36	<.001	—	0.21	—
Care recipients						
CCM → QoL	0.53	7.68	<.001	0.58	0.57	0.50
IC → QoL	0.25	2.74	.006	—	0.08	—
SS → QoL	0.18	2.01	.04	—	0.05	—

a QoL: quality of life.

b CCM: content creation and management.

c IC: information and communication.

d Not applicable.

e SS: safety and security.

### Dyadic Analysis (APIM)

**Figure 4.**
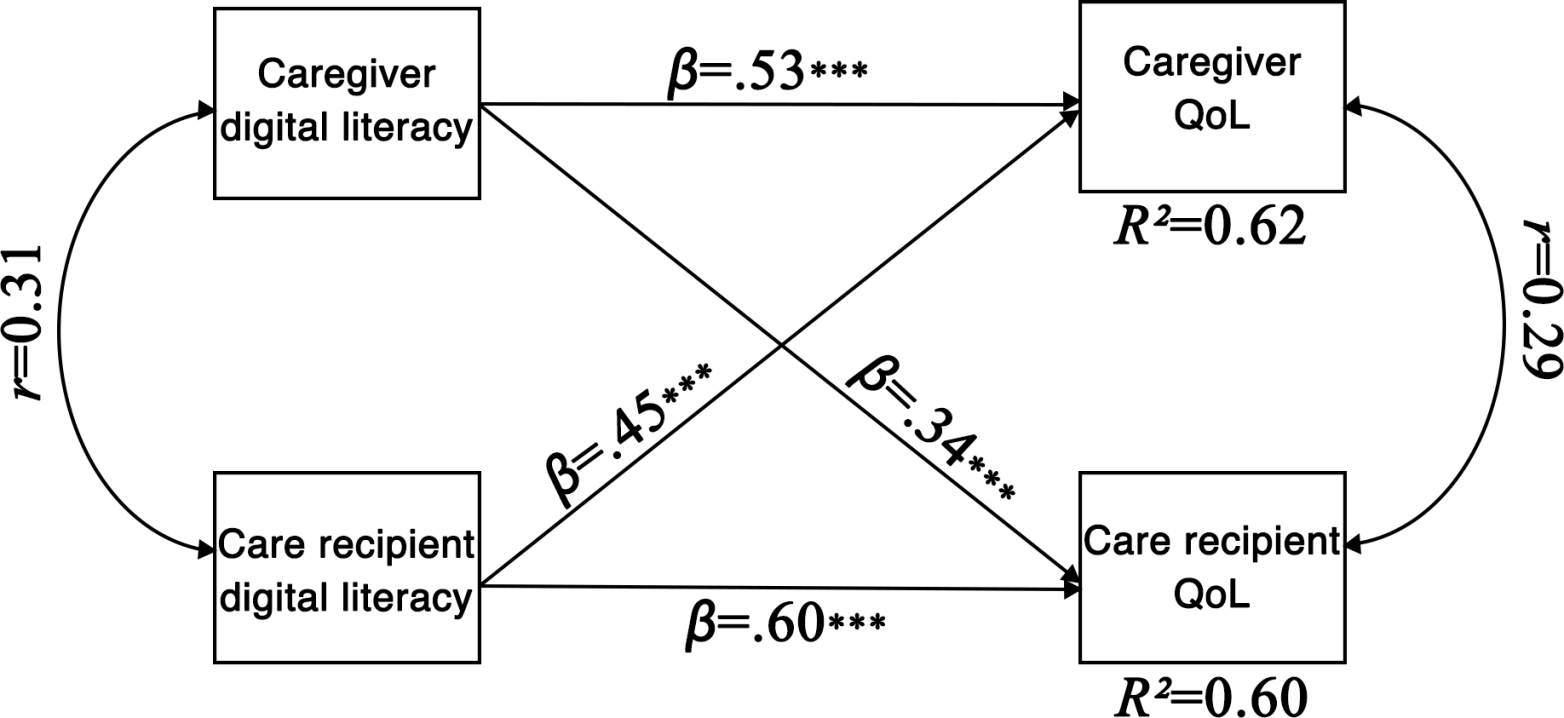
Basic actor-partner interdependence model of digital literacy predicting quality of life, showing standardized path coefficients (*β*), correlations (*r*), and explained variance (*R*^2^). N=72 dyads; QoL: quality of life. *P<.05, **P<.01, ***P<.001.

The basic APIM was estimated for 72 dyads using MLR estimation with full-information handling of missing data. Observed composite scores were used for caregiver and care recipient DL and QoL. The model included actor and partner paths for both caregivers and care recipients, allowing for correlated predictors and residuals between dyad members. All 4 APIM paths were statistically significant. Actor effects were positive for both caregivers (*β*=.53, *P*<.001) and care recipients (*β*=.60, *P*<.001). Partner effects were also significant, from caregiver to care recipient (*β*=.34, *P*<.001) and from care recipient to caregiver (*β*=.45, *P*<.001). Predictors were moderately correlated (*r*=0.31, *P*=.004). The residual correlation between caregiver and care recipient outcomes was small but significant (*r*=0.29, *P*=.045). Variance explained was substantial, with *R*²=0.619 for caregivers’ QoL and *R*²=0.595 for care recipients’ QoL. These *R*² values represent the combined variance explained in each outcome by both actor and partner effects. Figure 4 shows the basic APIM with standardized path coefficients and explained variance. Full statistical results are presented in Table 6.

**Figure 5.**
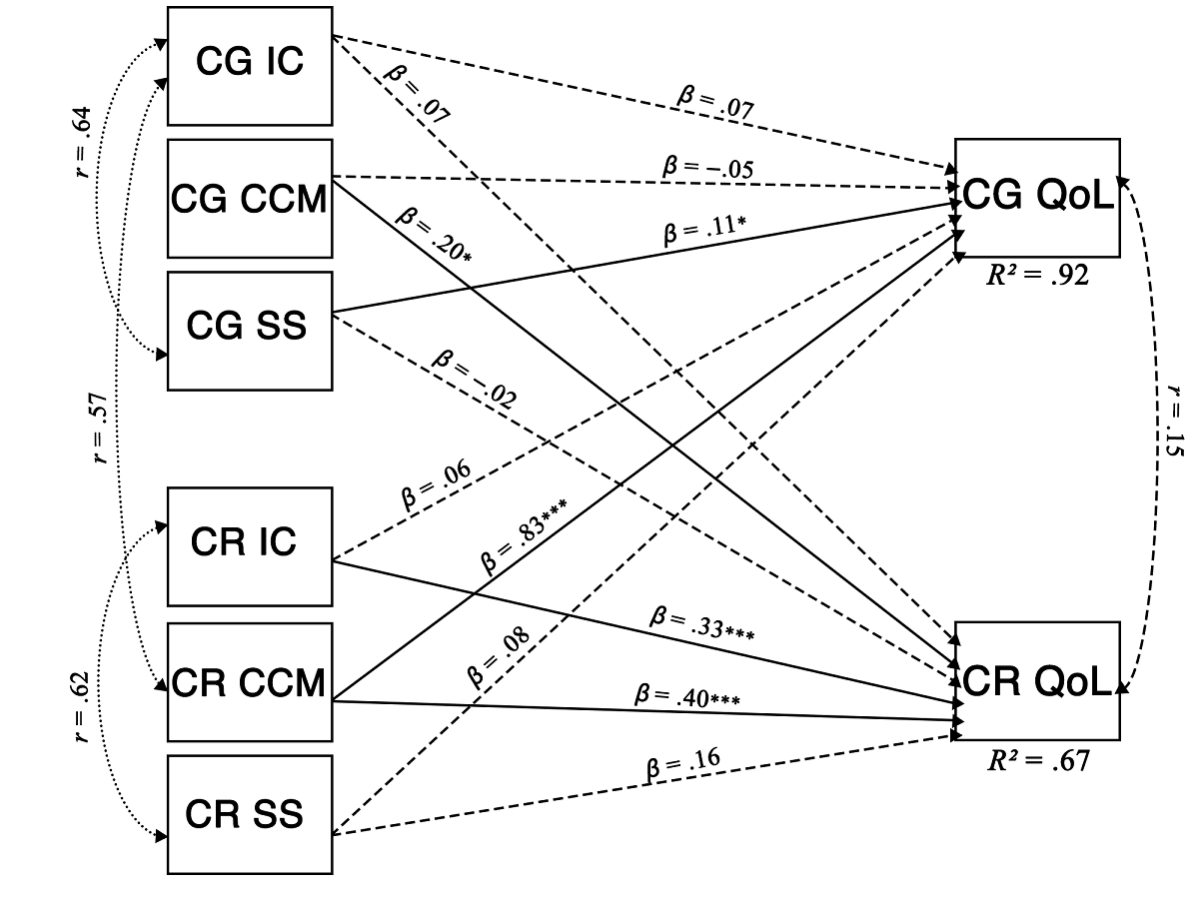
Multivariate actor-partner interdependence model of digital literacy dimensions predicting caregiver and care recipient QoL, showing standardized path coefficients (*β*) and explained variance (*R*^2^). N=72 dyads. Solid arrows indicate significant paths (*P*<.05); dashed arrows indicate nonsignificant paths; dotted double-headed arrows represent significant correlations among predictors. *P<.05, **P<.01, ***P<.001. CCM: content creation and management; CG: caregiver; CR: care recipient; IC: information and communication; QoL: quality of life; SS: safety and security.

In the multivariate APIM, caregiver and care recipient QoL were simultaneously regressed on 3 dimensions of DL (IC, CCM, and SS) for both members of the dyad. For caregiver QoL, a significant actor effect was observed for caregiver SS (*β*=.11, *P*=.022, 95% CI 0.02‐0.20). Other caregiver DL dimensions were not significant predictors. Among partner effects, care recipient CCM strongly predicted caregiver QoL (*β*=.83, *P*<.001, 95% CI 0.75‐0.91), whereas the other recipient dimensions were nonsignificant. The predictors explained 92% of the variance in caregiver QoL (*R*²=0.92). Model diagnostics indicated high intercorrelations among predictors, suggesting that the large *R*² primarily reflects shared variance rather than independent predictive effects. For care recipient QoL, significant actor effects were found for IC (*β*=.33, *P*<.001, 95% CI 0.15‐0.51) and CCM (*β*=.40, *P*<.001, 95% CI 0.21‐0.59). The effect of SS was positive but did not reach significance (*β*=.16, *P*=.072). Among partner effects, caregiver CCM significantly predicted care recipient QoL (*β*=.20, *P*=.047, 95% CI 0‐0.40), while other caregiver dimensions were nonsignificant. The predictors explained 67% of the variance in care recipient QoL (*R*²=0.67). Figure 5 shows the multivariate APIM with standardized path coefficients and explained variance. Full statistical results are presented in Table 7.

**Table 6.** Estimates from the actor-partner interdependence model of digital literacy predicting quality of life (N=72 dyads).

Path^a^	Role	*B* (SE)	*Z*	*P* value	*β*/*r* (95% CI)
CG^b^ DL^c^ → CG QoL^d^	Actor (CG)	0.55 (0.09)	6.00	<.001	*β*=0.53 (0.37-0.68)
CR^e^ DL → CG QoL	Partner (CR→CG)	0.56 (0.12)	4.68	<.001	*β*=0.45 (0.28-0.61)
CR DL → CR QoL	Actor (CR)	0.87 (0.15)	5.75	<.001	*β*=0.60 (0.45-0.75)
CG DL → CR QoL	Partner (CG→CR)	0.40 (0.11)	3.82	<.001	*β*=0.34 (0.16-0.51)
CG DL ↔ CR DL	Predictor correlation	45.60 (17.93)	2.54	.004	*r*=0.31 (0.10-0.52)
CG QoL ↔ CR QoL	Residual correlation	25.46 (14.81)	1.72	.045	*r*=0.29 (0.01-0.58)

a Estimator: maximum likelihood robust.

b CG: caregiver.

c DL: digital literacy.

d QoL: quality of life.

e CR: care recipient.

**Table 7.** Multivariate actor-partner interdependence model estimates of digital literacy dimensions predicting quality of life (N=72 dyads).

Outcome and path^a^	Role	*B* (SE)	*Z*	*P* value	*β* (95**%** CI)
CG^b^ QoL^c^					
CG IC^d^ → CG QoL	Actor (CG)	1.17 (1.02)	1.15	.25	0.06 (−0.04 to 0.17)
CG CCM^e^ → CG QoL	Actor (CG)	−0.88 (1.09)	−0.80	.41	−0.05 (−0.16 to 0.07)
CG SS^f^ → CG QoL	Actor (CG)	1.93 (0.86)	2.24	.02	0.11 (0.02 to 0.20)
CR^g^ IC → CG QoL	Partner (CR→CG)	1.12 (0.97)	1.16	.25	0.06 (−0.04 to 0.17)
CR CCM → CG QoL	Partner (CR→CG)	16.49 (1.06)	15.63	<.001	0.83 (0.75 to 0.91)
CR SS → CG QoL	Partner (CR→CG)	1.48 (1)	1.48	.14	0.08 (−0.03 to 0.18)
CR QoL					
CR IC → CR QoL	Actor (CR)	6.82 (2.10)	3.25	.001	0.33 (0.15 to 0.51)
CR CCM → CR QoL	Actor (CR)	9.20 (2.30)	4.00	<.001	0.40 (0.21 to 0.59)
CR SS → CR QoL	Actor (CR)	3.66 (1.96)	1.87	.07	0.16 (−0.02 to 0.34)
CG IC → CR QoL	Partner (CG→CR)	1.56 (2.24)	0.70	.49	0.07 (−0.13 to 0.27)
CG CCM → CR QoL	Partner (CG→CR)	4.26 (2.25)	1.98	.05	0.20 (0 to 0.40)
CG SS → CR QoL	Partner (CG→CR)	−0.38 (2.24)	−0.17	.87	−0.02 (−0.23 to 0.19)

a Estimator: maximum likelihood robust.

b CG: caregiver.

c QoL: quality of life.

d IC: information and communication.

e CCM: content creation and management.

f SS: safety and security.

g CR: care recipient.

Table 8 shows correlations among predictors in the multivariate APIM. Several strong associations were found, particularly between caregiver IC and caregiver CCM (*r*=0.57, *P*<.001), between caregiver IC and SS (*r*=0.64, *P*<.001), and between care recipient IC and SS (*r*=0.62, *P*<.001). These findings indicate moderate-to-strong associations among DL dimensions.

**Table 8.** Correlations among predictors in the multivariate actor-partner interdependence model (APIM; standardized estimates, N=72 dyads).

Path^a^	*r^b^*	SE	Z	*P* value
CG^c^ IC^d^ ↔ CG CCM^e^	0.57	0.08	7.04	<.001
CG IC ↔ CG SS^f^	0.64	0.07	9.22	<.001
CG IC ↔ CR^g^ IC	0.18	0.11	1.72	.09
CG IC ↔ CR CCM	0.56	0.08	6.94	<.001
CG IC ↔ CR SS	0.08	0.12	0.62	.53
CG CCM ↔ CG SS	0.53	0.08	6.53	<.001
CG CCM ↔ CR IC	0.12	0.12	0.98	.33
CG CCM ↔ CR CCM	0.46	0.08	5.67	<.001
CG CCM ↔ CR SS	0.19	0.11	1.68	.09
CG SS ↔ CR IC	0.15	0.10	1.47	.14
CG SS ↔ CR CCM	0.62	0.06	10.71	<.001
CG SS ↔ CR SS	0.13	0.13	1.01	.31
CR IC ↔ CR CCM	0.34	0.09	3.80	<.001
CR IC ↔ CR SS	0.62	0.06	10.99	<.001
CR CCM ↔ CR SS	0.25	0.10	2.47	.01

a Estimator: maximum likelihood robust.

b Reported values are standardized (STDYX) correlations among predictor variables in the multivariate APIM.

c CG: caregiver.

d IC: information and communication.

e CCM: content creation and management.

f SS: safety and security.

g CR: care recipient.

### Sensitivity Analyses

Three sensitivity analyses confirmed the robustness of FIML-based findings. Among 72 dyads, 47 (65%) dyads had complete data, 12 (17%) dyads had missing care recipient QoL only, 7 (10%) dyads had missing caregiver QoL only, and 6 (8%) dyads had both QoL variables missing. Little MCAR test (*χ*²_8_=7.82, *P*=.45) indicated that data were missing completely at random, supporting the MAR assumption underlying FIML.

EM imputation showed minimal deviation from observed values. For caregiver QoL, EM-estimated mean was 64.38 (SD 13.96) versus the observed mean of 64.46 (SD 14.68), a difference of 0.08 points. For care recipient QoL, EM mean was 54.69 (SD 16.12) versus the observed mean of 55.27 (SD 16.34), a difference of 0.58 points.

Re-estimating the basic APIM using listwise deletion (n=47 dyads) produced results highly consistent with FIML (N=72 dyads), as shown in Table 9. All 4 APIM paths remained significant (all *P*<.001), effect directions were unchanged, and standardized coefficients differed by an average of only 0.04 units. The consistency across missing data approaches confirms that FIML estimates are robust and that substantive conclusions are unchanged.

**Table 9.** Comparison of actor-partner interdependence model parameter estimates using full-information maximum likelihood and listwise deletion (N=72 dyads)^a^.

Parameter	FIML^b^	Listwise^c^	Difference^d^
Actor effects			
Caregiver DL^e^ → Caregiver QoL^f^	0.53	0.52	0.01
Care recipient DL → Care recipient QoL	0.60	0.54	0.06
Partner effects			
Care recipient DL → Caregiver QoL	0.45	0.41	0.04
Caregiver DL → Care recipient QoL	0.34	0.39	0.05
Explained variance (*R*²)			
Caregiver QoL	0.62	0.65	0.03
Care recipient QoL	0.60	0.65	0.05

a All path coefficients are standardized (*β*). All *P*<.001 for both full-information maximum likelihood and listwise deletion models. The average absolute difference across all 6 parameters was 0.04 units, indicating high consistency between methods.

b FIML: full-information maximum likelihood using all 72 dyads (18 caregivers and 13 care recipients with missing QoL data).

c Listwise deletion: complete-case analysis using 47 dyads with no missing QoL data.

d Absolute difference between FIML and listwise deletion estimates.

e DL: digital literacy.

f QoL: quality of life.

## Discussion

### DL and QoL: Role-Specific Patterns

The results of this study showed that DL has a multidimensional and role-specific relationship with QoL. The pattern of this association differed between caregivers and care recipients. Among caregivers, only the dimension of “SS” was a significant predictor of QoL. This finding suggests that the ability to navigate online spaces safely and maintain privacy was associated with a greater sense of control [16,28] and higher reported QoL.

Among care recipients, all 3 dimensions were associated with higher QoL. The largest effect was for “CCM,” followed by “IC.” This pattern suggests that care recipients who actively produce and organize digital content or engage in online communication are not just passive consumers of information but are also more likely to report greater autonomy, social connectedness, and overall satisfaction [29,30]. The findings are consistent with the study by Kyaw et al [7] among Korean older adults, which found that higher DL was associated with better perceived health and more technological self-care behaviors.

The APIM showed that the effects between caregivers and care recipients were reciprocal but asymmetric. Care recipients’ CCM skills showed a strong partner effect on caregivers’ QoL, while similar skills in caregivers showed a smaller but significant partner effect on care recipients’ QoL. These results emphasize that DL is not a uniform skill but rather a multidimensional construct that operates through different yet interdependent pathways between members of the caregiving dyad. The models explained more than 50% of the variance in QoL, indicating strong and stable associations.

In psychological and behavioral terms, safety-related DL can be associated with better perceived outcomes for caregivers. This skill is linked to higher trust in online resources and lower exposure to digital risks. For care recipients, communication and content creation skills were associated with greater self-expression and social engagement, 2 key components of psychological and social well-being. Recent research has also shown that improving digital health literacy increases self-efficacy and health-promoting behaviors in both groups. Taken together, these findings suggest that DL should be viewed as a multidimensional and relational factor within caregiving contexts [12,42].

### Dyadic Interdependence Between Caregivers and Care Recipients

The present study showed a clear pattern of bidirectional interdependence between caregivers and care recipients in DL and QoL. In the APIM, both the actor and partner effects were significant; that is, each individual’s DL predicted not only their own QoL but also their partner’s QoL.

In the basic model, interaction effects were observed in both directions. In the multivariate model, the paths were asymmetrical: CCM skills of care recipients showed a stronger association with caregivers’ QoL, while similar skills in caregivers showed a smaller but significant association with care recipients’ QoL. In contrast, safety-related DL in caregivers was associated only with their own QoL and was not significantly associated with the other party’s QoL. This finding suggests that digital safety may contribute more to caregivers’ sense of competence and personal stability than to care recipients’ direct outcomes.

This interdependence can be seen as part of the social and behavioral bonds in caregiving relationships [43]. When care recipients have higher DL, especially in communication and content creation, they become more active in self-care and digital interaction. This was associated with higher caregiver QoL and strengthens mutual reciprocity. On the other hand, caregivers’ DL was associated with the perception of a safer and more supportive environment within online environments, which was linked to greater trust and confidence among care recipients.

Therefore, educational interventions should be 2-way and interactive, moving beyond solely individual-focused approaches; they should involve training that focuses on shared learning and collaborative use of technology [44,45]. In Asian societies, where the family is still the center of care, strengthening DL within caregiving dyads may improve mutual well-being and may help reduce the digital divide in older adults. Future programs should consider DL not simply as an individual skill but as a relationship-centered asset that enhances the QoL of both.

### Digital Divide and Socio-Contextual Factors

In this study, care recipients had lower levels of DL than caregivers. Although this difference was not statistically tested, it is likely to reflect the “gray digital divide” that has also been reported in both global and regional studies. Recent meta-analyses suggest that eHealth literacy in older adults is still below optimal levels. This issue is particularly evident among women, the very old, single individuals, and those living in lower-income countries [46]. Similarly, in a national study conducted in China, differences in education, economic status, and media attitudes led to significant variations in technology use [47]. These underlying inequalities could explain the DL gap observed in Malaysian caregiving dyads.

As shown in Table 1, a significant portion of care recipients 54% (39/72) reported weak internet access, which may contribute to the digital divide and impact their QoL. At the national level, studies from Malaysia have shown that cognitive impairment and low socioeconomic status reduce the likelihood of using digital tools. Gender, age, and education also play a role in this pattern [48]. Broader research in Southeast Asia has also highlighted specific cultural barriers, such as fear of making mistakes, reliance on family, and limited rural infrastructure, that limit older adults’ independence in technology use [49]. Therefore, improving internet access and digital infrastructure for older adults may help alleviate these challenges and reduce the burden on caregivers. Overall, the digital divide in the care context is rooted in cultural and social factors rather than simply individual ones. These inequalities may undermine older adults’ digital self-efficacy and reduce their online participation. They may also place a greater educational burden on caregivers. Therefore, to achieve equitable well-being, digital inclusion programs should be designed at the family and community levels.

### Cultural-Contextual and Policy Dimensions of DL in Malaysia

Digital inclusion among Malaysian older adults is not limited to individual skills alone. It is also shaped by cultural norms and national policies that determine how technology is used. In our data, care recipients had lower DL than caregivers, consistent with a common pattern in collectivist Asian cultures, where older adults rely on family for digital access and decision-making. Digital learning in this context is often achieved through intergenerational interaction rather than through self-learning [50-52]. This may explain why, in our model, care recipients’ digital competence was associated with higher caregivers’ QoL.

In policy terms, Malaysia’s National Aging Policy recognizes the role of technology in enhancing independence but has yet to consider DL and participation as part of active aging. Similarly, Malaysia’s Digital Economy Roadmap [21] focuses on innovation and competitiveness but does not address the specific needs of care recipients and informal caregivers. As a result, despite our findings showing that DL is associated with better outcomes for both groups, existing policies still view technology as an economic and individual phenomenon rather than a social and relational one. The results showed that caregivers’ SS skills strongly predicted their QoL. Therefore, national aging programs should incorporate targeted cyber-safety modules for caregivers. Results also showed a strong effect of content-creation and communication skills on care recipient QoL. Hence, community DL initiatives should include training for care recipients to create, manage, and communicate digital content. These targeted areas align more closely with the dyadic and multidimensional patterns observed in this study. Together, these dyad-level recommendations operationalize a “2-way digital inclusion” approach, in which digital skills are developed reciprocally across care partners to enhance shared well-being and participation.

### Policy and Practice

The findings of this study emphasize that DL is a multidimensional and bidirectional concept that is related to the QoL of both members of the caregiving couple. The observed effects highlight 2 priority areas for intervention: caregivers’ SS skills and care recipients’ information, communication, and content-creation abilities. These results indicate that DL interventions should be dimension-specific and relationship-oriented.

Research by Dong et al [24] suggests that theory-based training programs, delivered in brief face-to-face or blended formats, can improve self-efficacy and digital health literacy.

In Malaysia, this approach could be incorporated into the “MyAgeing Digital Literacy Modules” [53], which align with the EDLQ domains. Integrating such modules into Pusat Aktiviti Warga Emas (PAWE) centers [54] and the Digital Rakyat platform [21] may strengthen caregivers’ cyber-safety practices and enhance care recipients’ content-creation competencies. This integration is likely to be more effective when supported by intergenerational guidance and real-time assistance. These efforts can complement broader national initiatives, including MyDIGITAL [21] and the Healthy Ageing 2030 plan [8], by promoting equitable and relationship-centered digital participation.

### Limitations and Future Directions

The cross-sectional design of this study limits the ability to infer causality and examine behavioral changes over time. Longitudinal dyadic studies are needed to establish how changes in one member’s DL may causally influence changes in the other’s QoL over time.

Moreover, our use of a composite QoL score, while methodologically necessary for the APIM framework and empirically supported by very high intercorrelations among WHOQOL-BREF domains (*r*>0.85), precluded examination of domain-specific effects. Future research with larger samples could employ separate APIM models for each QoL domain to explore whether DL dimensions differentially predict physical, psychological, social, or environmental QoL.

In terms of participant screening, our reliance on a single-item subjective memory complaint screen to ensure reliable self-report may not have adequately excluded participants with mild cognitive impairment, particularly among care recipients aged 75 years and older. While this pragmatic approach was necessary for feasibility in a community-based survey, the absence of objective cognitive screening means that some participants with undetected cognitive impairment may have been included, which could affect the reliability of self-reported DL and QoL assessments.

Although the multivariate model explained a high percentage of the variation in caregivers’ QoL (*R*²=0.92), this should be interpreted with caution. The high correlations between the different dimensions of DL (Table 8) suggest that part of this value may be attributed to the overlap of the constructs rather than the independent effect of each dimension.

As this study used purposive sampling and was conducted only in urban areas of Klang Valley, the generalizability of the findings to care recipients and informal caregiver populations in semiurban or rural areas of Malaysia is limited. Klang Valley differs from many other states in terms of DL, internet access, and socioeconomic conditions; therefore, the patterns obtained are not necessarily representative of the entire country.

Furthermore, the Malay translation of the EDLQ was reviewed by a bilingual academic with expertise in gerontology for linguistic appropriateness, but formal forward-backward translation protocols and comprehensibility pretesting were not conducted. Although both English and Malay versions were offered based on respondents’ language preference, the sample size did not permit measurement invariance testing to confirm equivalence across language versions. Future work with larger samples is needed to confirm measurement consistency across languages and respondent groups.

Future research should be conducted with more diverse samples from different regions to increase generalizability. Longitudinal or intervention studies could also shed more light on the causal pathways and lasting effects of digital education. Additionally, designing bidirectional and dimension-based programs with a focus on SS for caregivers and communication and content creation for care recipients can better demonstrate the mechanisms of digital empowerment. Finally, attention to digital equity and cultural adaptation is essential to improve access for less advantaged groups.

### Conclusions

In summary, this study demonstrated that DL is a multidimensional and bidirectional factor that is associated with QoL in informal caregiving relationships. Considering both actor and partner effects, the results revealed that caregivers’ safety skills and care recipients’ communication and management abilities were jointly associated with the well-being of both parties. Integrating bidirectional and dimension-based interventions into Malaysia’s national digital health and aging strategies can expand digital inclusion, reduce technological inequality, and may improve the experience of care. Future studies with longitudinal and educational designs can demonstrate how digital empowerment may enhance sustainable well-being in increasingly digital societies.

## Supplementary material

10.2196/86561 Multimedia Appendix 1 Scatter plot demonstrating variable distinctness.

## Abbreviations

APIM: actor-partner interdependence model
CCM: content creation and management
DL: digital literacy
EDLQ: Everyday Digital Literacy Questionnaire
EM: expectation-maximization
FIML: full-information maximum likelihood
IC: information and communication
MLR: maximum likelihood robust
QoL: quality of life
SEM: structural equation modeling
SRMR: standardized root mean square residual
SS: safety and security
VIF: variance inflation factor
WHOQOL-BREF: World Health Organization Quality of Life-BREF
