# Supplementary figures and images for: Multidimensional Digital Literacy and Quality of Life Among Informal Care Dyads in Malaysia: Cross-Sectional Survey
Source: JMIR Aging. 2026 Apr 27;9:e86561. doi: 10.2196/86561 (PMC13124688; doi:10.2196/86561)

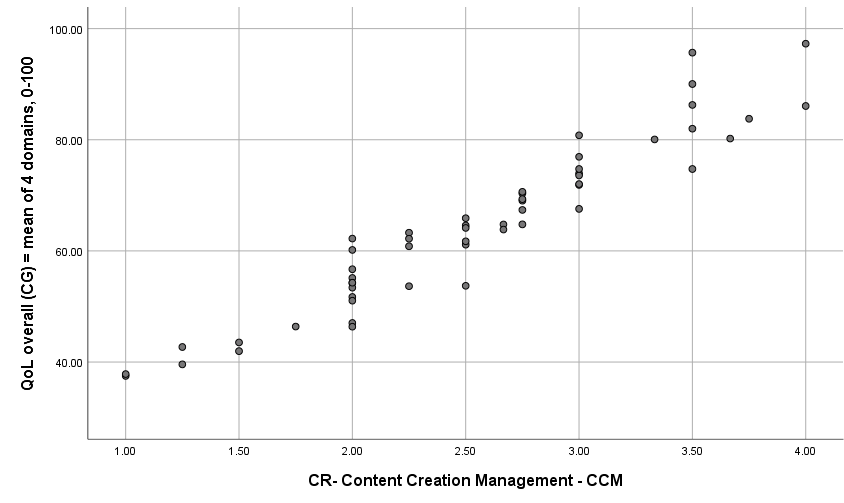

Supplement: Multimedia Appendix 1 [file aging-v9-e86561-s001.png]
